# A Progress Report and Roadmap for Microphysiological Systems and Organ-On-A-Chip Technologies to Be More Predictive Models in Human (Knee) Osteoarthritis

**DOI:** 10.3389/fbioe.2022.886360

**Published:** 2022-06-15

**Authors:** Mario Rothbauer, Eva I. Reihs, Anita Fischer, Reinhard Windhager, Florien Jenner, Stefan Toegel

**Affiliations:** ^1^ Karl Chiari Lab for Orthopeadic Biology, Department of Orthopedics and Trauma Surgery, Medical University of Vienna, Vienna, Austria; ^2^ Faculty of Technical Chemistry, Vienna University of Technology, Vienna, Austria; ^3^ Ludwig Boltzmann Institute for Arthritis and Rehabilitation, Vienna, Austria; ^4^ Department of Orthopedics and Trauma Surgery, Medical University of Vienna, Vienna, Austria; ^5^ Veterinary Tissue Engineering and Regenerative Medicine Vienna (VETERM), Equine Surgery Unit, University of Veterinary Medicine Vienna, Vienna, Austria

**Keywords:** osteoarthritis, organs-on-a-chip, microphysiological system (MPS), roadmap, disease modeling, alternative methods to animal testing

## Abstract

Osteoarthritis (OA), a chronic debilitating joint disease affecting hundreds of million people globally, is associated with significant pain and socioeconomic costs. Current treatment modalities are palliative and unable to stop the progressive degeneration of articular cartilage in OA. Scientific attention has shifted from the historical view of OA as a wear-and-tear cartilage disorder to its recognition as a whole-joint disease, highlighting the contribution of other knee joint tissues in OA pathogenesis. Despite much progress in the field of microfluidic systems/organs-on-a-chip in other research fields, current *in vitro* models in use do not yet accurately reflect the complexity of the OA pathophenotype. In this review, we provide: 1) a detailed overview of the most significant recent developments in the field of microsystems approaches for OA modeling, and 2) an OA-pathophysiology-based bioengineering roadmap for the requirements of the next generation of more predictive and authentic microscale systems fit for the purpose of not only disease modeling but also of drug screening to potentially allow OA animal model reduction and replacement in the near future.

## Introduction

**FIGURE 1:**
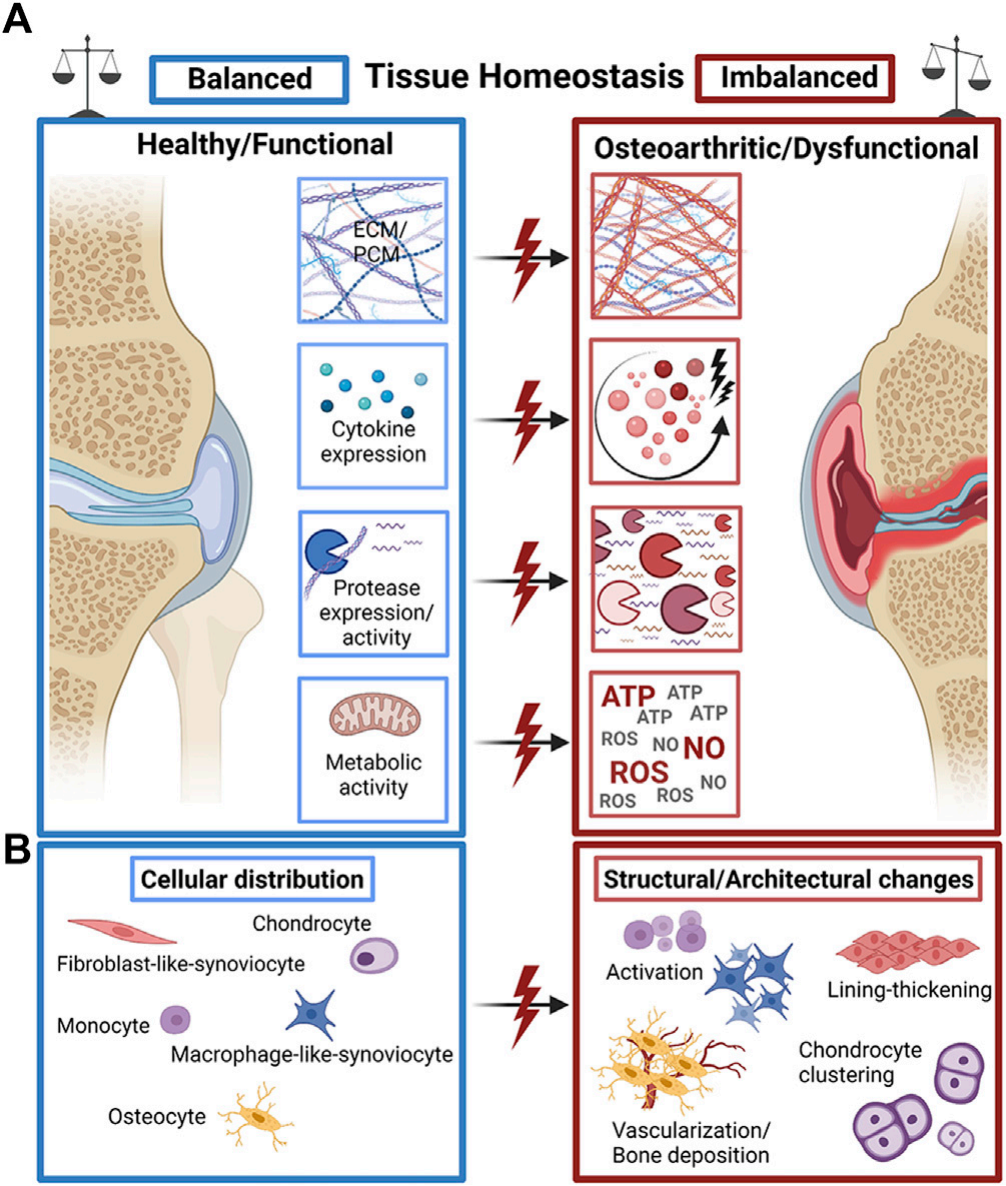
The fine line of tissue homeostasis in OA. **(A)** A plethora of structural and biochemical tissue factors such as extra- and pericellular matrix (ECM/PCM) biosynthesis, cytokine expression, protease expression and metabolic activity of cells guide the dysfunctional progression of OA joint tissues. **(B)** Changes in the distribution, occurrence, and activity of a variety of tissue-specific cell types mediate the structural decline of OA joint tissues with characteristic pathophysiological cell activation and proliferation, synovial lining thickening, and cell clone formation/clustering as well as pathological hypervascularization and bone deposition (calcification). Created with permissions from Biorender.com.

Osteoarthritis (OA), a chronic degenerative joint disease associated with substantial morbidity, disability, and reduced quality of life is the most common musculoskeletal disease affecting approx. 240 million people worldwide (March et al., 2016). Although OA is characterized by cartilage degeneration, inflammation, (premature) cartilage ageing, chondrocyte senescence, and phenotypic transitions (dedifferentiation and hypertrophic differentiation of chondrocytes), it is a disease of the entire joint (see Figure 1 for an overview), affecting all articular tissues because of their physical and functional association (Loeser et al., 2012). Current treatment strategies are only palliative and have little impact on the progressive degeneration of articular cartilage (Barry and Murphy, 2013). Driven by the unmet therapeutic need to reduce or reverse disease progression by either drugs or regenerative tissue engineering approaches, translational disease models for OA are key for the study of disease mechanisms, refinement of diagnostic methods, development of intervention strategies as well as identification of potent and effective disease-modifying therapeutic agents. The current review aims to outline the recent progress in OA modeling *in vitro* using advanced three-dimensional on-a-chip approaches and to provide a summary of essential aspects of the articular microenvironment and OA pathophysiology as the basis for a technological roadmap for the development of disease-relevant articular and OA joint tissue models in the second part of this review.

## The (Patho-)Physiological Articular Microenvironment as Blueprint for More Disease-Relevant *In Vitro* Osteoarthritis Models

### The Complex Physiology of a Joint

Throughout the human body, the three distinct types of skeletal joints are classified as either fibrous joints (synathroses), cartilaginous joints (amphiarthroses), or synovial joints (diarthroses) with their basic function of connecting skeletal parts such as the bone (Lawry and Bewyer, 2010; Zhang et al., 2015). Within the joint, the movements of the bones may be sliding, angular, and external or internal rotation. Among these types, synovial joints are the most complex structures with seven subclasses and a variety of mechanical functions (Lawry and Bewyer, 2010). The joint capsule is filled with synovial fluid (SF) and the synovial membrane continuously extends from the periosteum to the perichondrium secreting molecules such as lubricants into the joint cavity. Articular cartilage provides a smooth yet resilient surface for sliding between bone structures. Ensconced within the extracellular matrix of cartilage is a sparse population of chondrocytes (approx. 2% of the total volume of adult articular cartilage) as the sole resident cell type adapted to the low oxygen levels in its environment. Chondrocytes are phenotypically stable, maturationally arrested differentiated cells that maintain tissue homeostasis by synthesizing a very low level of matrix components to replace damaged matrix molecules, thereby preserving the structural integrity of the cartilage matrix (Goldring et al., 2011; Gilbert et al., 2021). Within their ECM, chondrocytes are surrounded by a narrow (2–4 μm thick) pericellular matrix (PCM) that is both biochemically and biomechanically (Young’s modulus 23–59 kPa) distinct from the ECM (Young’s modulus ≈500 kPa) and together with the ensconced cells is referred to as a chondron (Mow and Guo, 2002; Guilak et al., 2005; Gao et al., 2014; Chery et al., 2021). The extra- and pericellular matrix zones transmit the depth-dependent dynamic mechanical stimuli, comprising a combination of compression, hydrostatic pressure, shear stress, osmotic stress, and tensile strain, to the mechanosensitive chondrocytes, which in turn adjust cartilage metabolism depending on the magnitude, frequency, strain rate and nature of the applied load (Natenstedt et al., 2015; Gilbert et al., 2021; Statham et al., 2021). The bidirectional reciprocity in cartilage mechano-signaling enables chondrocytes to sense load application, including altered loading patterns, and in turn adjust matrix composition in response to mechanical cues. In this context, the PCM is pivotal in modulating the mechanical environment of the chondrocyte and regulating mechanotransduction in chondrocytes by transmitting biomechanical, biophysical, and biological signals between the ECM and chondrocytes (Mow and Guo, 2002; Gao et al., 2014; Chery et al., 2021). Based on the collagen fiber arrangement, distinct zonal chondrocyte phenotype, the density of proteoglycans, and expression of zone-specific markers, mature articular cartilage can be structurally and functionally divided into three distinct zones being the superficial (tangential) zone, the middle (transitional) zone, and the deep (radial) zone. A perpendicular tidemark region integrates the hyaline cartilage layer with the underlying calcified cartilage and subchondral bone (Bonnet and Walsh, 2005; Chauffier et al., 2012; Candela et al., 2016; Broeren et al., 2019). The subchondral bone is important for cartilage homeostasis as the stiffness of the underlying subchondral bone severely affects chondrocyte mechanosignaling (Carter et al., 2004; Zhen et al., 2021).

Reflecting on what has been mentioned earlier, successful recapitulation of an authentic joint environment *in vitro* needs to take many factors into account including general anatomical and structural as well as cellular, molecular, and biophysical properties.

### The Synovial Fluid is More Than Just an Ultrafiltrate

Having established on the overall joint anatomy, mechanics, and function, the role of intra-articular pressure (IAP) and the role of synovial fluid will be briefly outlined in the following. As hyaline cartilage is avascular, aneural, and alymphatic, synovial fluid and adjacent tissues provide its nutrient and oxygen supply and waste removal *via* loading-dependent transport through the ECM (pore size 2–10 nm) (Chahine et al., 2005), resulting in an oxygen gradient of 9%–2% and an osmolarity of 350–450 mOsm (Brighton et al., 1971; Urban et al., 1993; Rajpurohit et al., 1996; Sieber et al., 2020). Synovial fluid is a viscoelastic ultrafiltrate of plasma through the semipermeable synovial membrane supplemented with macromolecules secreted by synoviocytes (Levick and McDonald, 1995; Freemont, 1996; Sabaratnam et al., 2005; Blewis et al., 2007). Due to the plasma ultrafiltration, which allows proteins to cross only to a limited extent, physiological SF total protein (TP) concentration is approximately 25%–35% of the plasma protein concentration, while the glucose and electrolyte concentrations are similar to plasma (Weinberger and Simkin, 1989; Gobezie et al., 2007; Ritter et al., 2013). In healthy knee joints, the synovial fluid pressure is similar to the negative subatmospheric IAP of around −3 mmHg (Jayson and St Dixon, 1970; Blake et al., 1989) and stabilizes the whole joint keeping tissue portions in place. During exercise, the biomechanical forces on anatomical knee structures can almost triple and consequently lead to a rise in IAP above the capillary perfusion pressure (Kutzner et al., 2010) as well as shear force generation of around 20–30 dyn/cm^2^ (Tirtaatmadja et al., 1984; Hlaváček, 1995; Levick and McDonald, 1995; Schett et al., 2001). In turn, this impedes synovial perfusion *via* blood vessels and results in a more hypoxic environment during activity (Geborek et al., 1989). A rise in IAP as well as hypoxia can be also observed in OA patients. In addition, fluid pressure turbulences caused by fluid shear at the endothelial cell surface can promote inflammatory escalation (Albarrán-Juárez et al., 2018).

### Thoughts on the Mechanosensitive Synovial Membrane

The synovial membrane, which produces and maintains the specialized physical, cellular, and biochemical synovial environment, consists of two distinct layers: the synovium intima or lining, which is comprised of secretory fibroblast-like synoviocytes (FLS, 70–90% of the total cell population) and macrophage-like synoviocytes (MLS), and the underlying synovium subintima or sublining with an extensive system of lymphatics for clearance of transported molecules (Xu et al., 2003; Blom et al., 2004; Blewis et al., 2007; Hirschmann et al., 2007; Kiener et al., 2010; Zhang et al., 2019; Onuora, 2020). Both synoviocyte types are mechanosensitive and mechanoresponsive and exposed to a dynamic environment of mechanical stimuli including fluid- and contact-induced shear stress (Estell et al., 2017; Han et al., 2020; Thomson and Hilkens, 2021). Indeed, biomechanical stimuli, such as fluid-induced shear stress have been shown to influence FLS biosynthesis and modulate the effect of cytokines on FLS production of cartilage degrading enzymes (Estell et al., 2017; Han et al., 2020; Thomson and Hilkens, 2021). Similarly, mechanical cues are reported to guide macrophage activation and polarization as well as macrophage-chondrocyte cross-talk and to act as an immunomodulatory stimulus for macrophages (Estell et al., 2017; Han et al., 2020). Consequently, fluid pressure, fluid shear, as well as turbulences within the joint microenvironment can significantly influence the physiology and pathological progression of many musculoskeletal cell types including synoviocytes, endothelial cells, and chondrocytes.

This means that reengineering an authentic biophysical niche of a joint must consider many biophysical principles to create an *in vivo*-like environment for *in vitro* cell cultures.

### Soluble Tissue Crosstalk Contributes Many Vital Factors in Osteoarthritis Onset and Progression

Overall, the plethora of tissue types with distinct physical, mechanical, cellular, biochemical, and structural properties are conflating to create the complex microenvironment of a joint. This simple fact already teases that a single tissue-centered view cannot be a successful strategy to investigate a multifactorial musculoskeletal disease such as OA. The pathogenesis of OA is multifaceted, involving mechanical, cellular, and molecular processes, inflammation, metabolic dysfunction, and epigenetic modifications, and is orchestrated by cellular crosstalk of chondrocytes, synovial macrophages and fibroblasts, osteocytes, and infiltrating leukocytes, as well as alterations in the extracellular matrix (ECM) of articular tissues and synovial fluid composition (Loeser et al., 2012; Sellam and Berenbaum, 2013; Raman et al., 2018). OA can occur as a result of a variety of predisposing factors such as age, mechanical injury, genetics, gender, metabolic dysfunction, and obesity that incite a cascade of pathophysiological events within articular tissues (Loeser et al., 2012; Barboza et al., 2017). Irrespective of the initiating factors, the pathological progression of OA follows a consistent pattern (Goldring et al., 2011), indicating that a common molecular pathway [i.e., canonical NF-kB pathway (Marcu et al., 2010; Pichler et al., 2021, 2022)] links the biochemical and biomechanical processes that underlie the onset and progression of OA. Cartilage ECM debris caused by age-related wear or trauma is released into the synovial microenvironment activating synovial macrophages, synovial fibroblasts, and chondrocytes to produce inflammatory and catabolic mediators, which in turn disrupt cellular homeostasis and the balance between matrix synthesis and degradation in both tissues. Eventually, this creates a vicious cycle of tissue inflammation and breakdown (Barry and Murphy, 2013; Barboza et al., 2017). The normally quiescent chondrocytes, become activated and undergo a phenotypic shift characterized by cell proliferation, cluster formation, increased production of both extracellular matrix proteins and matrix-degrading enzymes, and hypertrophic differentiation (Barry and Murphy, 2013; Barboza et al., 2017). Exposure to inflammatory and oxidative mediators also enhances premature stress-induced senescence and ageing of chondrocytes resulting in an accumulation of senescent cells in the superficial layer of the articular cartilage. These cells in turn secrete a variety of inflammatory cytokines and matrix-degrading proteases linked to a senescence-associated secretory phenotype (SASP) (Bonnet and Walsh, 2005), which influences cell plasticity and propagates senescence and inflammation in surrounding cells and tissues. As an additional contributor, the subchondral bone is a source of inflammatory mediators implicated in clinical OA pain, hypertrophic differentiation of chondrocytes, and the degradation of the deep layer of cartilage, and it is involved in the abnormal distribution of stress on the bone-cartilage interface secondary to sclerosis and remodeling of the subchondral bone (Sellam and Berenbaum, 2013; Boris Chan et al., 2015; Aho et al., 2017; Hügle and Geurts, 2017). Recently, also adipose tissue as well as the synovial membrane are gaining more and more attention as significant contributors to the overall degradative and inflammatory biochemical microenvironment.

Building from the fact that this joint milieu is orchestrated by intricate cross-talk of a variety of different joint tissues, the inclusion of more sources of a pro and antiinflammatory mediator can and will shed more light on the contribution and mode of actions of individual tissues during onset as well as the progression of OA.

### Alterations in Matrix Compositions Guide Dysfunctional Biomechanics, Mechanosignaling, and Cell Activation

Another global factor of OA is the alteration of matrix biosynthesis and pathological matrix remodeling of a variety of tissues including cartilage, synovial membrane as well as subchondral bone. The ECM is key in mechanosignaling and mechanosensory regulation of matrix biosynthesis as well as pro and antiinflammatory processes. It has to be noted that the PCM as the closest cell interface is not a unique feature of chondrocytes even though most reported. PCM changes also correlate with loss of mechanotransduction activity in aging bone (Hagan et al., 2020), as well as fibrotic malformation of adipose (Divoux et al., 2010) and synovial tissue (Watson et al., 2011). Downregulation of matrix constituents such as perlecan and collagen VI further reduces PCM stiffness, which correlates with clone formation in the vicinity of cartilage defects (Foldager et al., 2014; Zelenski et al., 2015). OA alters the stiffness of the PCM (Zelenski et al., 2015; Danalache et al., 2019). These compositional changes can alter cell volume and morphology (Hall, 2019), TGF-β activation (Zhen et al., 2021) and proinflammatory cytokine and protease expression (Candela et al., 2016). An amplification of pathological mechanosignaling-related processes is further enhanced by overexpression of integrins due to mechanical overloading (Lucchinetti et al., 2004). Potentially, alterations in cell metabolism caused by dysfunctional matrix mechanosensing as described earlier further contribute to a more hypoxic synovial fluid environment that *via* hypoxia-induced oxidative stress lead to alterations in cell metabolism and activity (e.g., anaerobic glycolysis of chondrocytes, overproliferation, an increase of matrix biosynthesis, higher susceptibility to proinflammatory molecules, etc.) (Fermor et al., 2007; Mobasheri et al., 2017; Munjal et al., 2019). To give justice to the ultrastructural inadequacies of joint tissues in OA, *in vitro* models that claim to recapitulate matrix-related aspects should not be one-sided and focus on just a single aspect of matrix biology. The synergistic interplay between matrix biosynthesis, mechanosensation and cells as the three main pillars of dysfunctional matrix mechanobiology in OA must be accounted for in OA for the entire set of joint tissues to overcome the limitations of the current research approaches.

Overall, all the above physiological and pathobiological considerations at the tissue, cellular and molecular levels must be considered vital to develop an authentic microenvironmental niche. The implementation of these bioengineering parameters will in turn improve the predictiveness and authenticity of OA *in vitro* models. This is also evident as decades of cause-effect *in vitro* research analysis, i.e., proinflammatory cytokine secretion after a molecular OA stimulus has not resulted in any significant break-through innovation in disease therapy and tissue regeneration.

## *In Vitro* Technologies to Model the Cellular and Microenvironmental Complexity of Osteoarthritis

It is well-known nowadays that traditional two- dimensional (2D) culture models fail to replicate proper cell-cell and cell-matrix interactions necessary to mimic disease pathophysiology and are further limited by aberrant cell morphology, polarity, gene expression, and overall cell phenotype. Due to the lack of translational power, two-dimensional (2D) models are being progressively replaced by three-dimensional (3D) culture systems. Pellet, tissue explant, and micromass cultures more accurately mimic the native microenvironment found in musculoskeletal as well as any other tissue type. Explant models provide native tissue architecture and *in vivo-*like cell composition and thus feature the most authentic and “natural” microenvironment. Explant mono- and cocultures of equine (Haltmayer et al., 2019; Anderson et al., 2020) and porcine (Ding et al., 2014; Vernon et al., 2014) as well as human tissues (Topoluk et al., 2018; Dolzani et al., 2019) have been used to elaborate on the structure-function relationship between tissue architecture, the extracellular matrix composition and cell phenotypes in OA pathophysiology, but they are difficult to standardize due to the large interindividual variation. To improve standardization and thus comparability of data, pellet and micromass cultures, with and without natural (e.g., alginate, hyaluronan, collagen) or synthetic [e.g., polylactic acid, poly(ethyelene glycol)‐terephthalate] hydrogel-based scaffolds, have been developed (Smeriglio et al., 2015; Mouser et al., 2020). Thus, pellet cultures allowed, for example, to investigate the impact of disease-promoting factors, such as galectins, on ECM degradation *via* MMPs in the 3D context (Pichler et al., 2021, Pichler et al., 2022). Indeed, in 3D hydrogel culture, chondrocytes achieved near-native gene expression, chondral metabolism, and ECM turnover (Smeriglio et al., 2015) although the mechanical modulus of currently available hydrogels in the kPa magnitude range does not match the compression modulus of 9–13 MPa of healthy human cartilage tissue disks (Roberts et al., 1986; Cloyd et al., 2007; Guo et al., 2017). Moreover, 3D OA cartilage constructs in a poly(ethylene glycol)‐chondroitin sulfate hydrogel scaffold also exhibited pathological alterations in matrix biosynthesis such as decrease of COL2A1 expression, reduced glycosaminoglycan (GAG) content, and loss of compressive construct modulus (kPa) consistent with native OA samples when challenged with an inflammatory stimulus. Similarly, synovial micromass technologies comprising synovial fibroblasts, CD14^+^ monocytes and CD68^+^ macrophages (macrophage-like synoviocytes) demonstrated near-native cell composition and inflammatory response of the synovial intimal layer with increased proinflammatory cytokine expression, loss of antiinflammatory M2 macrophage phenotype, and synovial membrane hyperplasia when challenged with a proinflammatory stimulus (Broeren et al., 2019). Furthermore, 3D cocultures of synovial fibroblasts and endothelial cells have been established to model the contribution of synovial neoangiogenesis (Maracle et al., 2017), and even more complex 3D tricultures (e.g., including chondrocytes, synovial fibroblasts, and macrophages) modeled pathological chondrocyte activation and cartilage destruction in arthritis (Peck et al., 2018). Overall, primitive homo and heterotypic 3D culture techniques have given important insights into the complex structure-function relationship that influences matrix biosynthesis as well as the activity of any cell type within the joint tissue microenvironment.

### Biomechanical Complexity is the Key to Improve *In Vitro* Osteoarthritis Models

**FIGURE 2:**
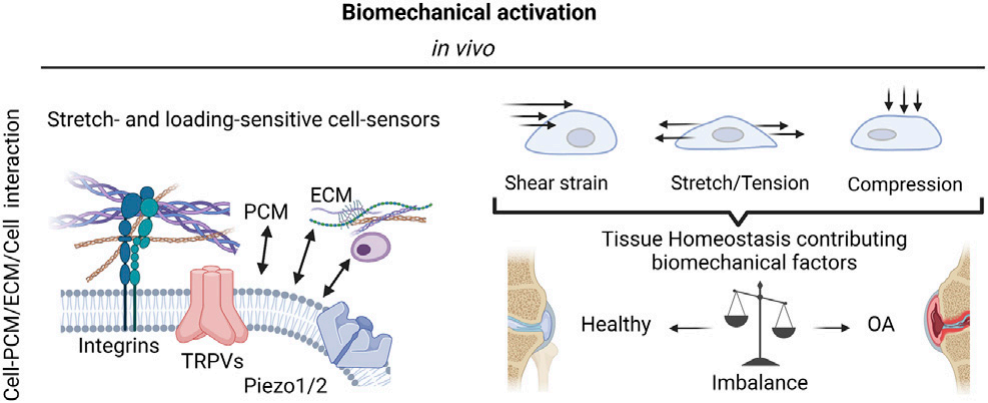
The complexity of biomechanical activation. The mechanosensitivity of knee-joint tissues and resident cells is mediated by integrins as well as stretch- and loading-sensitive cell-bound ion channels (mechanosensors) and include a variety of transient receptor potential (TRP) channels, and other mechanosensitive ion channels such as Piezo1 and Piezo 2. These mechanosensors in turn react to a variety of biomechanical forces such as fluid and mechanical shear strain, stretching, and tensile loading as well as compression to either promote joint tissue physiology or escalate the dysfunctional mechanobiology in OA *via* pathological mechanosignaling. Created with permissions from Biorender.com.

As outlined earlier, the physiology of a joint requires not only structural and cellular cues but also a variety of biomechanical forces for cells and tissues to maintain homeostasis. On the one hand, 3D printed zonal cell–scaffold structures aim to recapitulate physiological zonal cell distributions by either seeding cells directly on printed scaffolds or printing materials with encapsulated cells have been developed (Guo et al., 2017; Mouser et al., 2020). This creates better architectural control over the biomechanical properties such as matrix stiffness and hardness to recapitulate the anisotropy of material properties within even a single joint tissue type. On the other hand, the integration within mechanical bioreactors allows explants or tissue-engineered constructs to investigate the impact of fluid flow, compressive loading, and other important biomechanical forces (see Figure 2) and to look into matrix catabolism, metabolic cell activation as well as inflammatory and nociceptive signaling (Fermor et al., 2002; Piscoya et al., 2005; Chauffier et al., 2012). Mechanical bioreactors have been used routinely to improve the chondrogenic microenvironment for stem cell-based systems as well as primary chondrocyte models (Fu et al., 2021). More authentic multidimensional actuation principles combining shear and compression can further approximate *in vivo* chondrocyte matrix biosynthesis as well as lubricin overexpression (Meinert et al., 2017). Simultaneous control over oxygen tension with feedback loops and nitrogen supply can further tune the topography of physiological matrix constituent deposition increasing GAG secretion to the superficial zones of a construct with upregulation of bulk COL2A1 and ACAN expression (Tekari et al., 2020). Overall, research on conventional 3D cultures demonstrated the complex relationship between structural, biochemical as well as biomechanical cues in dysfunctional tissue homeostasis found in OA.

### Organs-On-A-Chip can Provide More Refined Tissue Architecture, Cellular Distribution and Tissue-like Mechanobiology

**FIGURE 3:**
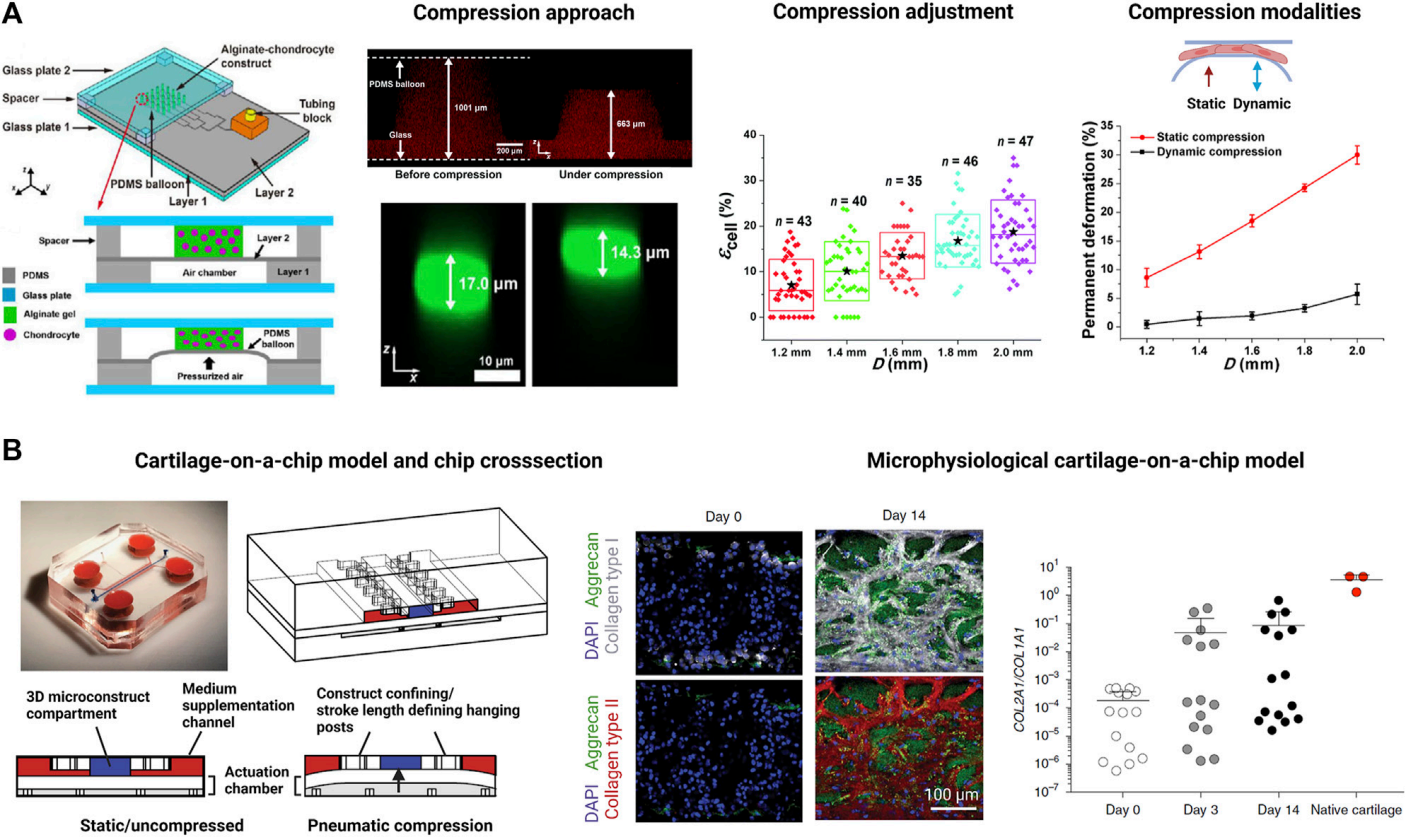
Examples for biomechanically actuated joint-on-a-chip systems. **(A)** A microfluidic pneumatic-actuation device transmitting multiple mechanical stress conditions on chondrocyte-laden alginate hydrogel to study deriving mechanisms of bone growth. *Via* a pneumatic channel network, pressurized air deflates a silicone (PDMS) balloon to compress chondrocytes. For instance, hydrogel compression of 34% in the *z* direction (red fluorescence) leads to a compression of individual chondrocytes within the hydrogel by 16% (green fluorescent cells). Increase of the diameter of the silicone balloon gradually increases cell loading by up to 30% under constant and cyclic loading conditions (see graphs on the right). Reproduced with permissions from (Lee et al., 2018). **(B)** Development of a cartilage-on-a-chip (left panel) where a 3D cartilage construct (blue compartment) enclosed by two medium supplementation channels (red highlights) is actuated by pneumatic compression of a deflectable membrane (white membrane). Physiological compression of 10% results in a native tissue-like matrix biosynthesis (center panel) comprising high levels of aggrecan (green fluorescence), collagen type I (white fluorescence), and collagen type II (red fluorescence). Analysis of the Collagen type II-to-I ratio which is used as an indicator for cartilaginous matrix approximates native cartilage after 14 days of biomechanical on-chip cultivation. Reproduced with permissions from (Occhetta et al., 2019).

**FIGURE 4:**
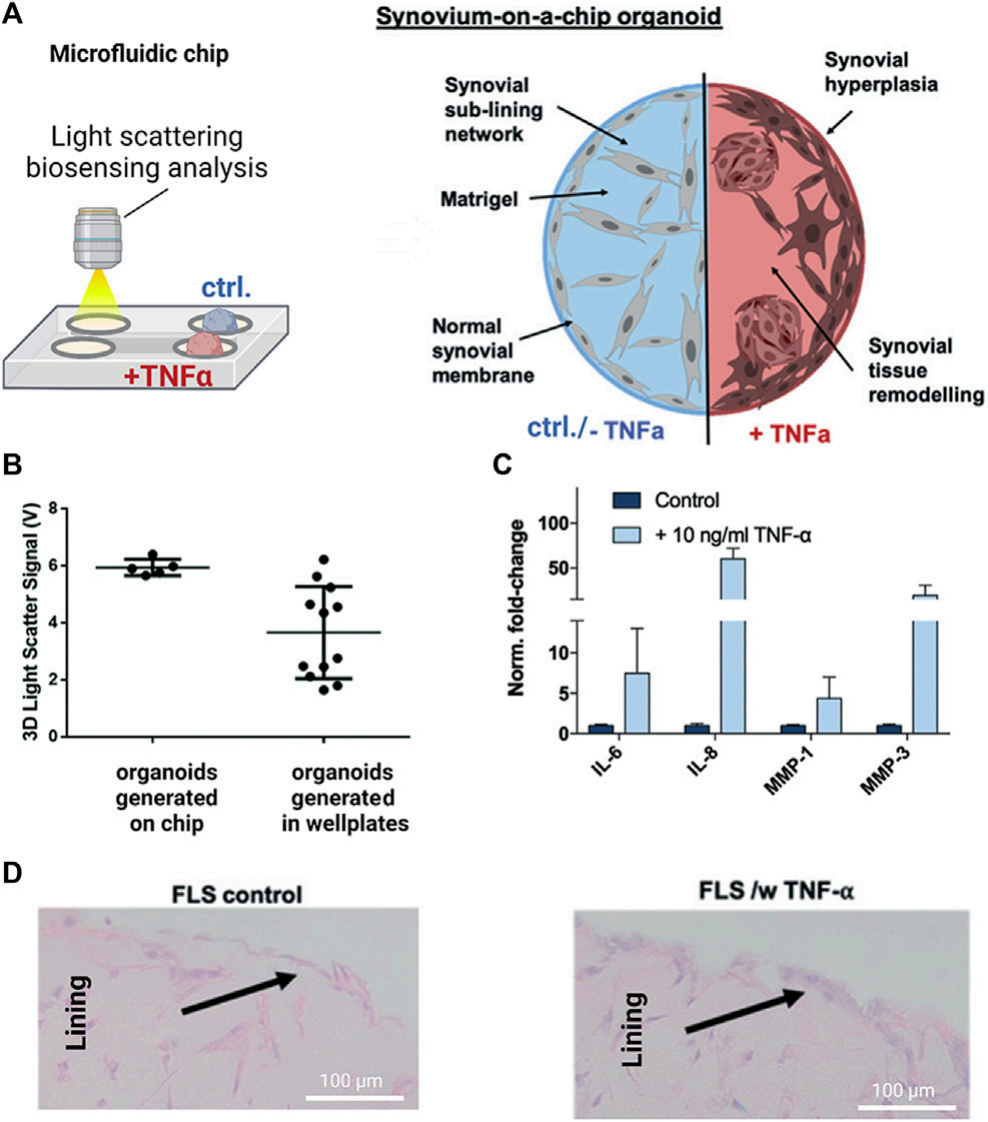
Noninvasive biosensor integration enables structural analysis of synovial on-chip organoids. **(A)** Light scattering measurements were combined with a human synovium-on-a-chip (top panels) to investigate structural alterations in rheumatoid arthritis (RA). **(B)** Light scatter analysis of on-chip generated untreated synovial organoids shows higher reproducibility than conventional synovial micromasses generated in microtiter plates while **(C)** retaining their ability to respond to TNF-α stimulation with upregulation of interleukins (IL-6/-8) and proteases (MMP-1/-3) **(D)** Synovial on-chip organoids showed synovial lining hyperplasia upon TNF-α stimulation characteristic for RA synovial intima. Reproduced with permissions from (Rothbauer et al., 2020).

**FIGURE 5:**
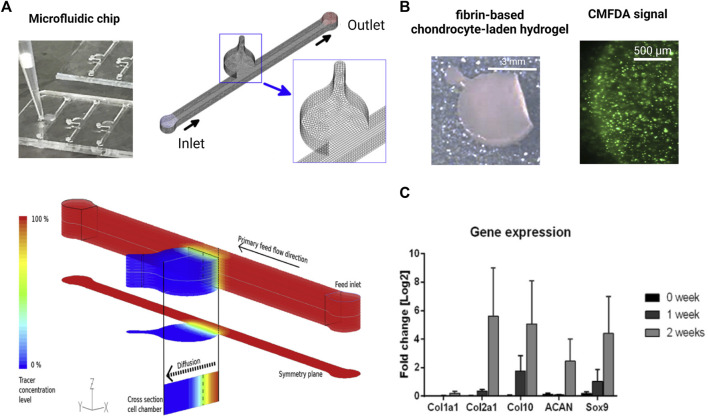
Biochip technologies for native tissue-like molecular gradients. **(A)** Macroscopic cartilage-on-a-chip system presenting native-like tissue-like molecule diffusion dynamics analyzed by fluid dynamic simulations. **(B)** Steep nutrient and metabolite gradients do not alter the high chondrocyte viability of chondrocytes (green fluorescent CMFDA assay) and showed **(C)** cartilage-specific molecular expression levels of matrix and chondrocyte-related genes analyzed by RT-qPCR over 2 weeks of cultivation. Reproduced with permissions from (Rosser et al., 2019).

As the most recent advancement in the palette of 3D culture techniques, organ-on-a-chip (OOAC) and microphysiological systems (MPS) aim for an even better recapitulation of a native tissue-like environment, tissue architecture and cell-specific responses relevant to OA modeling by combining stem cell or patient-derived primary cell-based models for cartilage and synovium (Fomby et al., 2010; Smeriglio et al., 2015; Tian et al., 2016; Li et al., 2017; Lin et al., 2019) as well as adipose (Loskill et al., 2017) and bone-like tissues (Mansoorifar et al., 2021; Nasello et al., 2021). In this context, microfluidic technologies can create an even more dynamic yet more controllable musculoskeletal disease microenvironment. Because OA has long been considered a cartilage disease, most microphysiological models of OA still focus on chondrocyte pathobiology. Analogous to traditional 3D cultures, microfluidic cartilage-on-a-chip approaches have demonstrated their ability to recapitulate near-native tissue-like conditions on a structural, architectural, and molecular level (e.g., morphology and matrix biosynthesis, and inflammatory signaling). Biomechanical actuation by compressive loading has also been demonstrated to be a critical parameter for biochip chondrocyte 3D cultures as shown in Figure 3A (Lee et al., 2018). Cell stimulation can be easily performed within biochips using integrated pneumatic deflectable actuator structures. Chondrocytes embedded in hydrogel can in turn be deformed in the presented approach up to 30% cell deformation for either static or cyclic compression routines with no alterations in chondrocyte health. In the same line, dynamic compressive loading in a physiological range (6–10%) improved *in vivo* like cartilage gene expression, while hyperphysiological compression around 30% loading triggered OA-like chondrocyte responses (Occhetta et al., 2019; Figure 3B). Overloading could significantly reduce aggrecan ACAN gene expression and Collagen II-to-I ratio while increasing gene expression levels for markers involved in chondrocyte hypertrophy and inflammation (i.e., collagen type X, MMP-13, IL-6, and IL-8). To integrate more natural molecule diffusion distances above 500 μm, musculoskeletal tissue models that feature single constructs with macroscopic dimensions have been established. A synovium-on-a-chip system combined with integrated sensors as shown in Figure 4 demonstrated the feasibility of time-resolved multiplex analysis schemes (Rothbauer et al., 2020). The study utilized light scattering to noninvasively probe dynamic synovial tissue-level responses when challenged with proinflammatory cytokines including synovial network architecture remodeling and organoid condensation altered by cadherin-11-mediated cell-cell adhesion. In addition, cartilage-on-a-chip systems as demonstrated by Rosser et al. (2019) can already well resemble cartilage hallmarks of the middle and superficial zone with proper chondrocyte morphology and gene expression while precisely controlling the molecule gradients within cartilage constructs due to the microfluidic flow (see Figure 5). Moreover, the authors used their miniaturized drug screening tool to recover cartilage-specific OA-like inflammation responses with a treatment. Notably, to further investigate and approximate *in vivo-*like tissue conditions, increase of microfluidic construct diameter and volumes by twofold increased collagen and glycosaminoglycan biosynthesis as well as the corresponding dynamic construct modulus (Tian et al., 2016). Both studies demonstrate well that the geometry and macroscopic dimensions of a construct influence the models’ diffusivity as well as topographical molecule gradients that in turn are vital to control parameters for organoid maturation and native tissue-like physiology.

**FIGURE 6:**
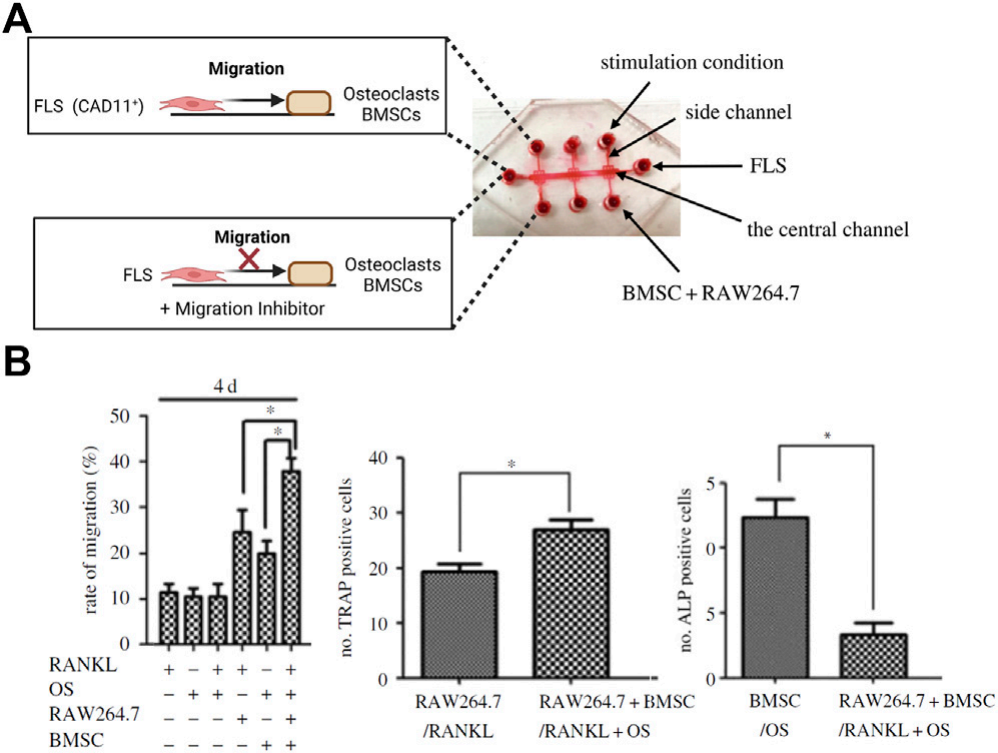
Compartmentalized organs-on-a-chip feature migration dynamics of an arthritic tissue microenvironment. **(A)** Using a triple culture biochip, the biochip model was used to analyze FLS migration towards a bone model (bottom panel) to simulate fibroblast invasion mechanisms in bone tissue. **(B)** The rate of synovial migration was highest for the triple coculture stimulated with Receptor Activator of NF-κB Ligand (RANKL; left graph). An increase in numbers of TRAP-positive catabolic osteoclasts and decrease of anabolic ALP-positive osteoblasts recapitulates *in vivo*-like processes happening during the synovial bone invasion and bone erosion. Reproduced with permissions from (Ma et al., 2018).

**FIGURE 7:**
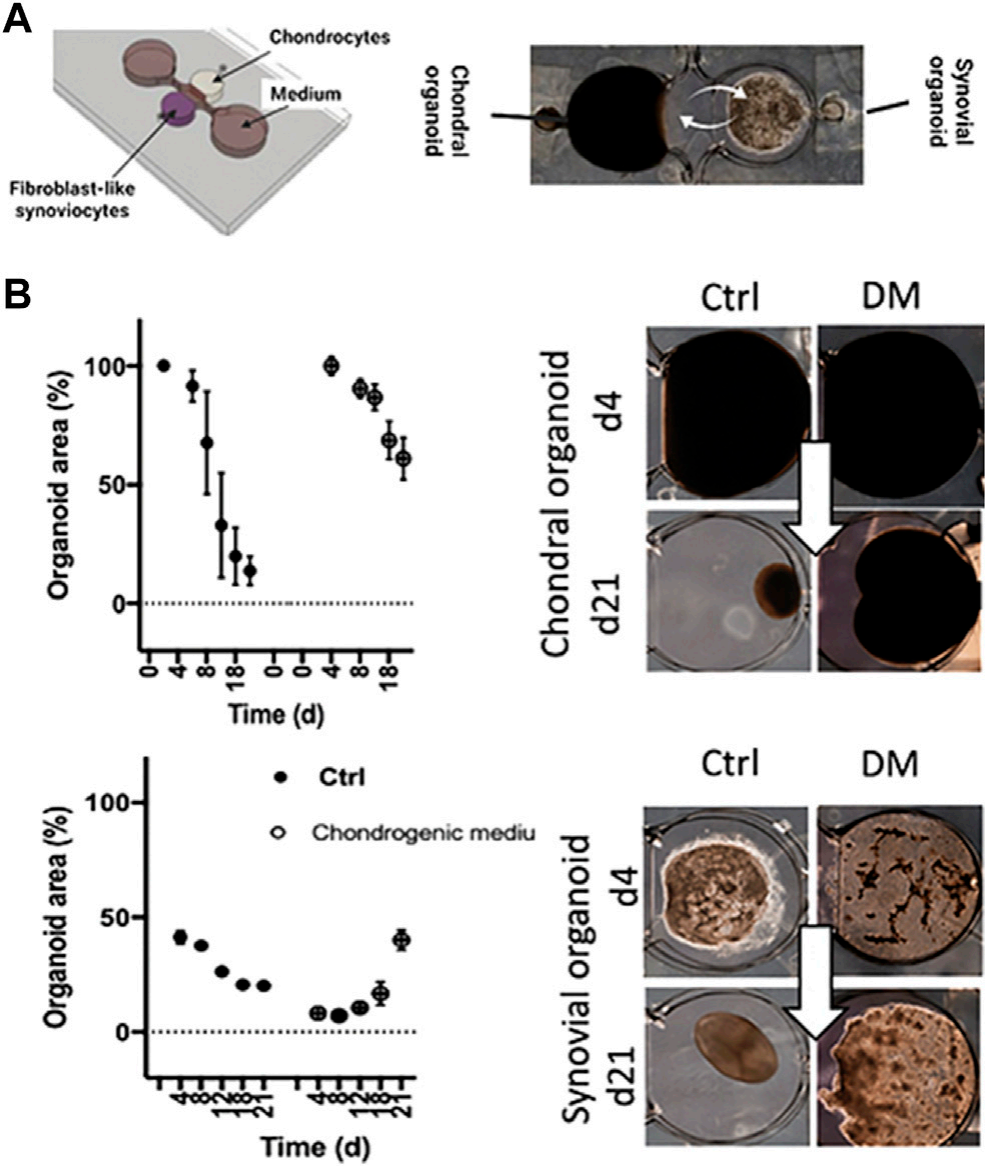
Tissue level crosstalk fosters an anabolic and antiinflammatory joint environment. **(A)** A millimeter-sized 3D coculture model recapitulating only the soluble synovial and chondral tissue-crosstalk was used as an RA model based on healthy cadaveric chondrocytes and RA patient-derived FLS embedded in 3D hydrogels. **(B)** Analysis of the potentially fibrotic effect of commercial differentiation medium (DM) on condensation analysis of chondral and synovial cocultures. Adapted with permissions from (Rothbauer et al., 2021).

**FIGURE 8:**
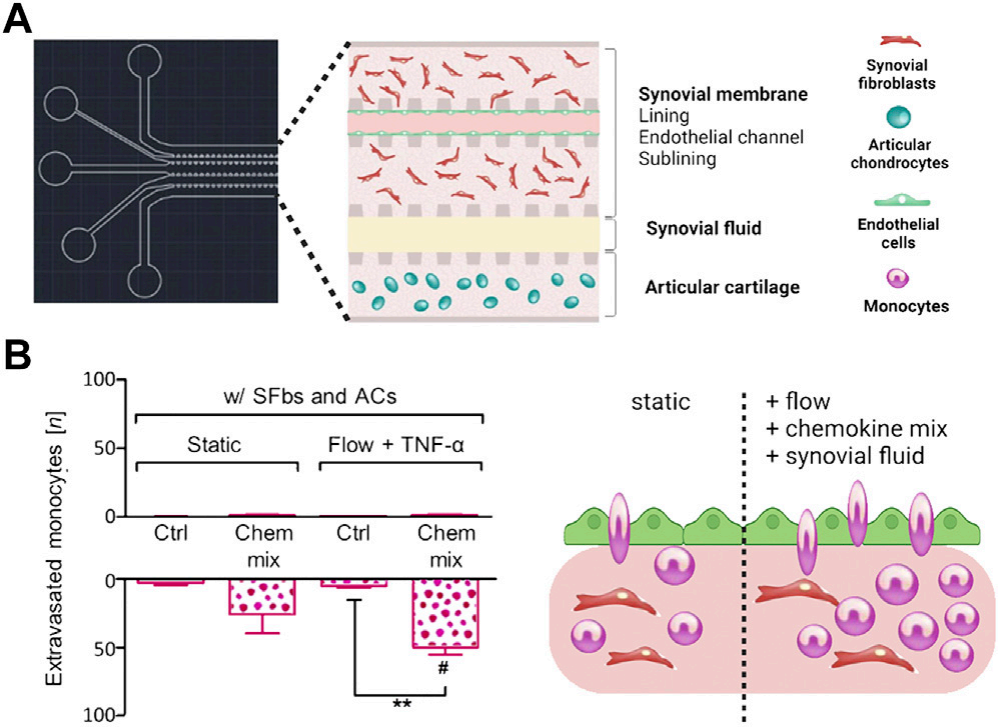
Flow regimes alter immune cell migration in more holistic *in vitro* joint tissue studies. **(A)** A multicompartment biochip with heterotypic triple cultures comprising articular chondrocytes (blue cells) and an endothelialized synovial membrane model (turquoise endothelium with red FLS) for monitoring of monocyte extravasation and tissue infiltration processes in OA synovial tissue. **(B)** The combination of flow with synovial fluid and chemokines increases the number of extravasated monocytes within the synovial tissue compartment. Reproduced with permissions from (Mondadori et al., 2021).

As outlined before, OA is considered a serious multifactorial disease affecting multiple tissue structures (Dieppe, 1999; Loeser et al., 2012; Andriacchi et al., 2020). Consequently, a deeper understanding of tissue communication is paramount to understand general as well as disease phenotype-specific mechanisms (Mobasheri and Batt, 2016; Mobasheri et al., 2019; Salgado et al., 2021). Combining cartilage-on-a-chip systems with other musculoskeletal tissues such as the synovium or subchondral bone has proven essential in providing a more holistic view of tissue-tissue interactions that may govern OA onset including inflammation, fibrosis, and degradation of joint tissues (Piluso et al., 2019). The impact of synovial secretoma on overall bone cell homeostasis was investigated using a coculture microsystem of human synoviocytes (i.e., SW982 sarcoma cells) with murine preosteoclasts (i.e., RAW264.7) and primary stem cell-derived osteoblasts (Ma et al., 2018). As shown in Figure 6, the migratory behavior analysis of activated synoviocytes towards osteoclasts in the bone compartment can potentially shed light on initial mechanisms of erosion. Integration of synovial with chondral compartments as shown in Figures 7A, B demonstrated that the soluble cell-cell communication by healthy synovial fibroblasts contributes to a more physiological chondrogenic microenvironment (i.e., round chondrocyte morphology with reduced cell activation and dedifferentiation) (Rothbauer et al., 2021). The addition of a chondrogenic differentiation medium induced a fibrosis-like catabolic synovial response with the disintegration of the synovial organoids. The inclusion of monocytes/macrophages, endothelial cells, and physiologically relevant fluid shear conditions within another chondro-synovial biochip shown in Figure 8A mimicked the synovial postcapillary venule. Monocyte chemotaxis and migration from the bloodstream into synovial tissue (Figure 8B) as a model for synovial immune cell infiltration was enhanced when TNF-α treatment was combined with the fluid flow (Mondadori et al., 2021). To demonstrate that nutrient and molecule gradients can be generated in macroscopic multiphasic constructs, 3D-printed multichamber bioreactors were used to bioengineer sophisticated anisotropic osteochondral architectures (Lin et al., 2014). A two-phasic construct was seeded with heterogeneously differentiated hBMSCs to develop prechondral and osseous phenotypes. Tissue-specific hallmarks included a tidemark-like region, anabolic gene expression, and matrix production. When stimulated with IL-1β, the model developed an OA-like tissue response including an expression decrease in chondral markers (e.g., SOX9, COL2A1 and ACAN) in the context of osteochondral tissue-level communication.

## Bioengineering Roadmap—Requirements for the Next Generation of OA Models for Human Disease Modeling and Drug Screening

For the next technological leap, we advocate a reverse engineering approach following the origins and strengths of organs-on-a-chip technology to recreate authentic organ or tissue-level function and architecture while deliberating on homo- and hetero-typic cell and tissue interactions and also the cellular identity and activation states that altogether regulate important anabolic and catabolic molecular but also structural aspects of tissue homeostasis and function during disease onset and progression.

### Mind the Joint Biomechanics and Mechanobiology

**FIGURE 9:**
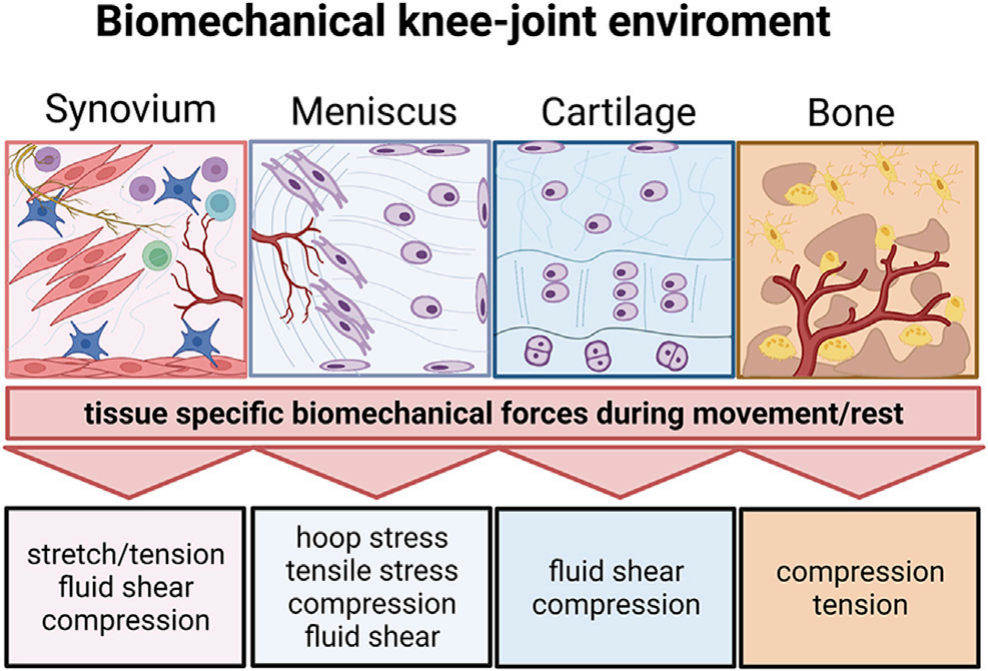
The biomechanical knee-joint environment matters. Synovial joints comprise a very diverse set of heterotypic tissue architectures including the synovium, meniscus, cartilage, and subchondral bone. Similar to their architecture, the respective biomechanical microenvironment during joint movement is tissue-specific and comprises distinct biomechanical principles. Created with permissions from Biorender.com.

In general, biomechanical cues including compression, shear, interstitial flow and hydrostatic and osmotic pressure can regulate pro or antiinflammatory responses in a variety of tissues and cell types (see Figure 9) highlighting the necessity for more complex and dynamic culture environments also *in vitro* (Li et al., 2018; Fahy et al., 2019). Mechanical stimuli provide a proper microenvironmental niche for *in vitro* disease models. Mechanosensory activation (e.g., Piezos, TRPs or integrins, etc.) during the osteoarthritic onset and progression is a critical, to date largely ignored, mechanosignaling aspect of microsystems, which must be closely investigated (Statham et al., 2021). Consequently, to improve existing models, we propose to apply anatomical and (bio)mechanical considerations for the next-generation microsystems. The structure-function relationship of an articular joint is very complex and multi-faceted and not only includes a variety of cellular and biochemical but also many important biophysical parameters. In addition to nutritional functions, the synovial fluid, as a common biofluid mediating tissue homeostasis and communication, has also an important biophysical and fluid-mechanical function. Because pathological conditions (i.e., the rise of IAP) can be biomechanically and microenvironmentally very similar to healthy exercising conditions, the selection of cells and tissues from disease origin will be critical for an authentic disease pathophenotype. Future joint-on-a-chip models must increase the controllability and precision of fluid-mechanical cues at the microscale. In combination with biomechanical loading, future microphysiological systems can offer better precision and control over spatio-temporal and regional multiparametric mechanical stimulations based on principles well established over two decades of microsystems engineering and microfabrication. In this line of thought, biomechanical cues within microfluidic joint-on-a-chip systems can be potentially further approximated to the biophysical complexity and anisotropy including more *in vivo*-like force orientation (Paggi et al., 2020) using a combination of multiple actuators working at various dimensional axes. Technical advancement of chip-based mechanostimulation from pneumatic to other actuation approaches will provide a better technological basis to create systems with higher throughput (Qian et al., 2020) including force-time analysis curves of human patients (Phinyomark et al., 2016; Kapri et al., 2021) as control input curve will provide cells with more biomimetic force patterns *in vitro*.

### Mind the Tissue Extracellular Matrix

**FIGURE 10:**
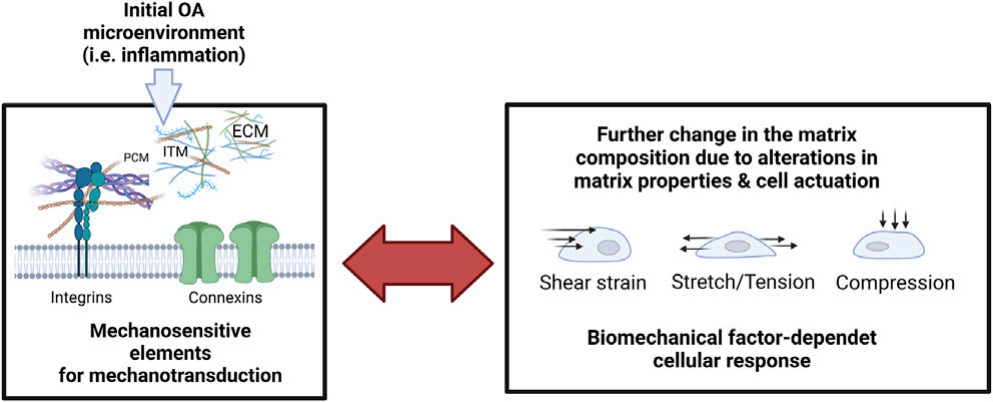
The reciprocity of cellular mechanosensation escalates the pathological response during inflammatory onset and progression of OA. Microenvironmental factors of the extracellular matrix (extracellular matrix, ECM; pericellular matrix, PCM; Interterritorial matrix, and ITM) are monitored by cell-bound mechanosensitive structures such as integrins and connexins that again mediate dysfunctional cell activity and pathological matrix biosynthesis. Pathological mechanosignaling in turn influences the cellular sensation towards shear strain, stretching, and compressive loading. Created with permissions from Biorender.com.

The inclusion of a near-native and tissue-specific composition of ECM components including collagens and proteoglycan fillers is key to generating the proper cellular microenvironment found in OA. Collagen fiber arrangement and morphology also affect how the ECM responds mechanically to compressive, shear, hydrostatic, osmotic, and tensile loading. Changes in the composition and stiffness of the extracellular interterritorial, territorial and pericellular matrix (see Figure 10) provide essential cues for ECM-sensitive mechanosensors such as integrins and also connexins to further escalate pathological matrix remodeling towards an OA phenotype. Consequently, future microsystems must consider topographic and regional control over matrix compositions and orientational properties to create a more native pathophysiological template for disease-relevant cell-matrix interactions. The PCM has a pivotal role in the bidirectional reciprocity of cartilage mechanosignaling and homeostasis. Moreover, PCM degeneration is one of the earliest events during OA onset, altering the stress-strain microenvironment of chondrocytes leading to aberrant chondrocyte mechanotransduction (Gao et al., 2014; Gilbert et al., 2021). Microphysiological systems must start to recreate PCM and ECM compositions and architectures that are seen in actual OA tissues. Novel microencapsulation techniques could be modified to generate chondron-like structures that simulate the microenvironment of chondrocytes (Li et al., 2019). Furthermore, to recapitulate the native niche, attention needs to be paid to the hydraulic permeability coefficient, which governs fluid movement in cartilage loaded in compression and is in turn related to the matrix pore structure, size, and connectivity (Mow and Guo, 2002; Jackson and Gu, 2009; Sophia Fox et al., 2009). Overall, matrix hydrogel systems that have the same origin as the target tissue will show the highest potential to trigger native-like cell and tissue responses for both stem cells as well as primary cell types alike. This is an important aspect that has been investigated for decades for repopulated decellularized organ matrices (Guyette et al., 2016; Ohata and Ott, 2020) but still needs to be implemented properly for the next generation of organs-on-a-chip and microphysiological systems. This will obviously exclude the use of highly artificial hydrogel and scaffold systems (e.g., Matrigel, GelTrex, Fibrin, Gelatin, PEG, etc.), that cannot provide the right matrix architecture and composition found in healthy as well as diseased human tissues. Concerning matrix zonation and fiber orientation control, optical and extrusion bioprinting already show great promise to create biomimetic templates that are fit for musculoskeletal engineering as the alignment of individual structures can be controlled in a layer-by-layer fashion (Rothbauer et al., 2022).

### Mind the Vascular and Lymphatic (Patho)Physiology

**FIGURE 11:**
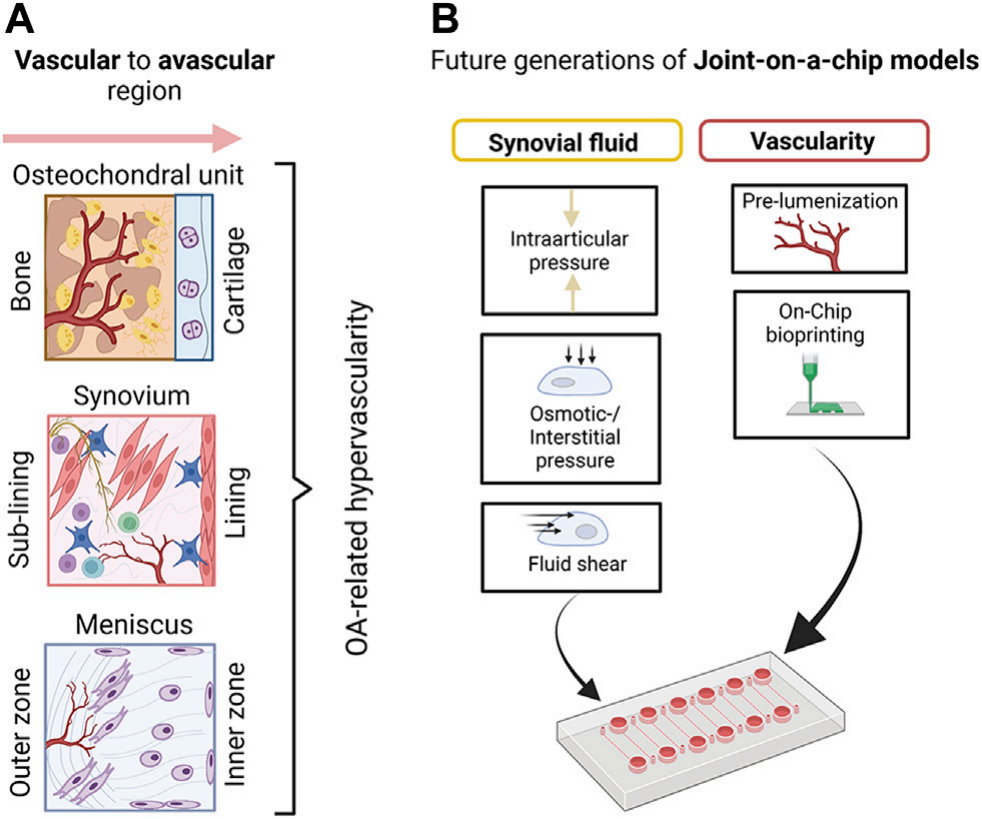
**(A)** A schematic overview of basic vascular transitions within musculoskeletal tissues which are affected by OA-related hypervascularization. **(B)** A proposed future strategy to model joint tissue-specific transitions with biomimetic vascularized joint-on-a-chip systems includes the creation of vascular blueprints/templates with bioprinting as well as the provision of more relevant biofluid properties (e.g., viscoelasticity of blood and synovial fluid surrogates) and fluid-dependent biomechanical principles including fluid interstitial, as well as intra-arcticular pressure. Created with permissions from Biorender.com.

As shown in Figure 11A, the general architecture of joint tissues often comprises a vascular region that transitions to an avascular region over multiple tissue layers or phases. Osteochondral tissue, synovium, or menisci are good examples of vascular-avascular transitions (i.e., blood—synovial tissue—synovial fluid). As indicated in Figure 11B, multiphasic systems can generate a range of molecular and biomechanical gradients as not only blood perfusion as a function of vascularization degree but also matrix composition and stiffness vary significantly even within individual tissue types (i.e., cartilage zones), which are all part of a physiological cellular and tissue microenvironment. Similar to the ambitions of the body-on-a-chip community to create microsystems that logically connect vascularized organ models (Ryu et al., 2015; Kratz et al., 2019), also joint-on-a-chip systems need to improve their vascular (as well as lymphatic) content using either prelumenized vascular blueprints/templates or vascular networks (Shi et al., 2014; Whisler et al., 2014; Knezevic et al., 2017; Bachmann et al., 2018) that form within a predefined tissue compartment or individual tissue zones guided by self-assembly and endothelial sprouting. For applications that are too complex for simple bifurcated lumen structures or even self-assembly, on-chip optical bioprinting can create even more biomimetic structures such as vascular beds at very high resolution (Grigoryan et al., 2019). Adding biomimetic tissue complexity can elaborate on the manifold relationship of vascular signaling and pathological architecture on overall joint tissue homeostasis for obviously fibrous tissues such as synovium, fat pads, and meniscus but also for osteochondral bone. These tissues are severely affected by vascular invasion and other pathological events involving bone resorption and osteophyte formation (Bonnet and Walsh, 2005; Suri et al., 2007; Hamilton et al., 2016). Potentially, the next generation of models should also consider better fluid components that recapitulate the viscoelastic, rheological, and biochemical properties of blood and synovial fluid which will also impact osmotic and interstitial pressure as well as fluid shear on mechanosensitive joint tissues. Also, the gaseous microenvironment provided by flow gradients will influence cell and tissue identity. However, hypoxia incubators and chambers can only adjust the entire microenvironment rather than generating a gradient by regional flow and gas control. In addition to variable tissue-specific flow conditions provided by microfluidic technologies, the application and integration of scavenging materials, vacuum degassing regions, or simply a natural adjustment of oxygen content by cell numbers *via* cell metabolism (Zirath et al., 2018; Sticker et al., 2019), as well as spatially-resolved oxygen feedback loops using sensor arrays can further improve microsystem control from a tissue perspective (Kratz et al., 2019).

### Mind the Cell Origin, Identity, and State

The maturity and origin of cells for 3D *in vitro* models constitute an enormous challenge that needs significant technological improvements. Culturing cells *in vitro* also includes their cultivation in an artificial, stimulative, and supplemented fluid environment, mainly with the aim to promote proliferation. Especially stem cells are often differentiated into joint tissue-specific cells and progenitor cells (Lin et al., 2014; O’Grady et al., 2019; Pirosa et al., 2021) for bone, cartilage, adipose, or connective tissue cells using differential cultivation protocols (Lin et al., 2019). Given the cell population heterogeneity of resident mature and progenitor cell types in native OA tissues (Stephenson et al., 2018; Cheng et al., 2021; Wang et al., 2021; Liu et al., 2022), stem cell approaches for future *in vitro* models must provide tissue-specific mature cell types with high phenotypic and genotypic authenticity in addition to a variety of heterogeneous progenitor cells. The direct and indirect crosstalk between various progenitor and adult cell subpopulations will in turn further advance the authenticity as well as the inflammatory responsiveness of the next-generation microsystems. Considering the physical alignment of tissue-resident cells, mature and progenitor cells can simply be adjusted by the cell mixing ratio within microfluidic compartments (Rothbauer et al., 2018); however, advances in bioprinting also for microfluidic organs-on-a-chip demonstrate new degrees of freedom and capabilities of cell deposition within 3D volumes (Rothbauer et al., 2022). Since OA is a disease of an adult population, the application of cells in early developmental stages (i.e., stem cells) to recapitulate the cellular composition of pathological adult tissues must be questioned critically. Even adult cell types show tissue-specific functions as demonstrated, e.g., for the differences between abdominal and infrapatellar tissue adipocytes in inflammatory M1-macrophage response (Barboza et al., 2017). Moreover, the *in vivo* hormonal tissue environment (Nevitt et al., 2001; Linn et al., 2012; Jin et al., 2017; Maghbooli et al., 2019) prior to cell isolation (Xue et al., 2018) alters the *in vitro* performance of patient-derived primary cells. This means that the authenticity of soluble biochemical cues such as hormones and potentially also a variety of other bioactive molecules including fatty acids, adipokines, and glycans found in mature tissue is paramount to recreating an authentic pathophysiological cell and tissue phenotype of OA (Toegel et al., 2009, 2010; Pabst et al., 2010). Considering that the prevalence of OA increases with age and is higher in women, especially after menopause (Phinyomark et al., 2016), cell donor choice is essential to achieve a disease-relevant and authentic model.

Another aspect to consider is the need for multiphasic and heterogeneous differentiation protocols including divergent differentiation and cultivation durations for the proper maturation of cells within 3D models. A first step to address this challenge is to generate growth factor gradients to tune the differentiation of bioprinted multiphasic tissue constructs (Lin et al., 2019; Pirosa et al., 2021). Nonetheless, with the increasing complexity of the microsystem, the spatial and temporal control of growth factor administration decreases. We thus propose to better use and employ liquid handling approaches such as concentration gradient generators and μ-valves for temporal and spatial separation of individual tissue and cell compartments for future models.

### Thoughts on the Final Engineering Tasks of Automation and Scalability

The requirements to successfully transfer this future biomimetic OA microsystem from an academic technology to its envisioned application in the pharmaceutical industry as a drug screening tool should already be considered in the ongoing design and development phase. From a technological standpoint, the integration and application of the currently available palette of integrated on-chip functions that organ-on-a-chip and microphysiological systems can offer still lags behind the evident cellular and molecular biological advancements improving the tissue-like architecture, individual cell phenotypes, gene expression profiles, as well as pro and antiinflammatory secretion. Over the last decade, a variety of technological improvements has been made to integrate on-chip functions within cell-based microfluidic systems to gain better control over the overall homo and heterotypic cell-cell interaction as well as the biophysical and chemical microenvironment (e.g., shear flow, loading, oxygen concentration, ECM compositions and stiffness gradients, nutrient supply and waste removal, etc. (Ribas et al., 2018; Rothbauer et al., 2018; Piluso et al., 2019; Sticker et al., 2019). Consequently, many principles that have been successfully reported for biomechanical (micro)bioreactors, cell-based microfluidics, and lab-on-a-chip systems including functional materials, degassers, microactuators as well as multicompartmental networks (Sticker et al., 2017; Piluso et al., 2019; Shabestani Monfared et al., 2020) have already shown great potential to also increase the capabilities of current organs-on-a-chip and microphysiological systems.

Even though many promising studies have combined industrial microfabrication technologies such as polymer hot embossing and electroplating (Novak et al., 2013), current academic approaches mostly lack the technology transfer from small academic production to large scale series production of complex organs-on-a-chip and microphysiological systems due to the ongoing strong academic dependence on poly(dimethyl)siloxane (PDMS) material. PDMS microfabrication is very affordable and straightforward and it offers great material advantages such as optical transparency, acceptable biocompatibility as well as gas transparency. Another plausible explanation for this limiting material selection is the fact that in most cases a standard 3–4 years research project does not exceed production batches of around a few 1,000 pieces including several design optimizations and iterations within a normal project lifetime. Consequently, fabrication methods for biomedical-grade hard polymers are still limited to specialized research groups due to infrastructural bias or budgetary limitations for both material pellets as well as injection tools that would provide industrial prototyping qualities for mass production. Notably, computer numerical control (CNC) micromachining or hot embossing of hard polymer slides would bridge the gap between soft lithography and injection molding. Nonetheless, scalable and high potential state-of-the-art technologies to improve the functionality of organs-on-a-chip and microphysiological systems may include well-established approaches found mostly for lab-on-a-chip and micro-total-analysis (µTAS) systems including integrated microvalves, micropumps, and gradient generators to control the automated administration of fluids at the picolitre scale (Unger et al., 2000; Frey et al., 2014; Tian et al., 2016; Rothbauer et al., 2019). To further improve sample throughput, a combination of gravity-driven pumpless bidirectional fluid handling technology (Sung et al., 2010; Esch et al., 2016) with robotics (Novak et al., 2020) may also be a high potential candidate. For models that require unidirectional pumping, this approach can also be modified to provide unidirectional fluid flow and shear when required (Wang and Shuler, 2018). Even more biomimetic flow conditions combining microsystems with bioprinting technologies as demonstrated by cardiac microsystems need to be considered also for a musculoskeletal vascular environment (Zhang and Larsen, 2017; Grigoryan et al., 2019).

To improve the high-content capabilities of currently available microsystems, a broad palette of micro and biosensors as well as inline and off-chip analysis schemes have been reported mostly as proof-of-concept studies (Kratz et al., 2019). Overall, the applicability of noninvasive monitoring approaches is high; however, any optical, electrical or chemical approach requires defined technological prerequisites including highly specialized measuring set-ups (e.g., electrical contacting, optical read-out positioning, specialized tables, etc.). This in turn reduces the manufacturability as well as integration potential with conventional incubator systems found in academia as well as industry labs. Moreover, the inclusion of a variety of cell- and tissue-specific analysis parameters that potentially drive the content of such a microsystem will impede the throughput capabilities of microsystems due to the necessity of printed circuit boards (PCBs), external multiplexing systems as well as a fair extent of electrical and/or optical fiber wiring to provide good measurement signal-to-noise ratio (SNR) signals. The synergy of noninvasive and dynamic analysis schemes with conventional destructive endpoint analysis technologies will allow more insights into the time-resolved response of bioengineered systems prior to the relative read-out at defined endpoints to better question aspects of cellular and donor variability and heterogeneity (Masaeli et al., 2016; Moura et al., 2019) as well as disease pathogenesis and tissue-specific pathomechanisms. As a final remark for this bioengineering road ahead, the necessity of artificial intelligence and deep learning (Hashemzadeh et al., 2021) to support faster data analysis and interpretation arise, since throughput and content as both throughput and content of our bioengineered systems will also potentiate the number of individual analysis parameters (e.g., secretion profile, mRNA expression, structural imaging, multidimensional cell morphologies, and interactions, etc.). Multi-parametric datasets can further benefit from potent high-content analysis schemes such as single-cell sequencing (Liu et al., 2022) and advanced mass spectrometry imaging (Rossiter et al., 2021; Vandenbosch et al., 2021). Finally, validation of microphysiological disease models must ideally be benchmarked against human data; however, no human patient data are available for the early stages of arthritic disorders. To tackle this challenge, species studies from which legacy data are available across any temporal range of pathogenesis stages will provide a good validation strategy for disease onset and progression validation (Jang et al., 2019; Marx et al., 2020).

## Concluding Remarks

Biomimicry of the pathological articular microenvironment should consider authentic tissue architectures, matrix compositions, cell heterogeneity, gene expression, and protein secretion (i.e., cell-cell interactions *via* soluble factors) in addition to general biomechanical as well as biochemical gradients found in human articular tissues to improve the capabilities and potential of the current disease models. Recent methodological advances in microfluidic technologies already provide better spatial and temporal control and heterogenic distribution over heterotypic cell populations, as well as biochemical and biomechanical traits with regard to conventional tissue-like models. Many key aspects that guide dysfunctional tissue homeostasis in the osteoarthritic environment *in vivo* still need to be implemented. Given the complexity of the articular environment, OA pathophysiology, and bioengineering approaches, an interdisciplinary approach will be needed to develop a disease-relevant *in vitro* model. To facilitate the necessary integration of recent technological advances and current knowledge on joint physiology and OA etiopathogenesis, we here draw a bioengineering roadmap to define key requirements that will significantly improve the *in vivo* relevance, predictability, and applicability of future chip-based disease models as drug screening tools. The current progress especially for joint-on-a-chip technology as disease models and drug screening tools in arthritis may not seem surprising at first glance because the technological progression follows advancements of previous *in vitro* models and approaches for arthritic diseases: RA-related studies focus mostly on synovial and immune contribution to catabolic processes such as secretion of catabolic mediators and mechanisms of bone resorption whereas OA-related studies survey predominantly the effect of degradative molecules but also proinflammatory mechanisms to affect cartilage as the key target tissue. The combination of cartilage and synovial cell populations with bone, vascular, or even autologous immune cell subpopulations of individual primary patient origin will show significant impact on how far we can advance joint multi-tissue coculture approaches in the future. Concluding all that has been mentioned earlier, a transdisciplinary effort is necessary to achieve the main aim of recreating native tissue-like models for musculoskeletal diseases. We need to appreciate and integrate all the lessons learned so far on joint tissue homeostasis and pathogenesis including joint biomechanics and tissue-level anatomical architecture, tissue-specific variations in the composition of the ECM, regional differences and neuralization and vascularity as well as the cellular origin, identity and activation states to achieve a model with authentic tissue-level responses relevant to OA. This will necessitate a cross-disciplinary effort combining regenerative medicine, tissue engineering, bioengineering, chemical engineering, mechanical engineering, and biomechanics in addition to cell and molecular biology to create a biotechnological leap forward. For the creation of fit-for-purpose technology applicable for industrial drug screening applications, the scalability of the model regarding analysis throughput and the content will further require close collaboration with mechanical engineering experts and industrial chip manufacturers.
